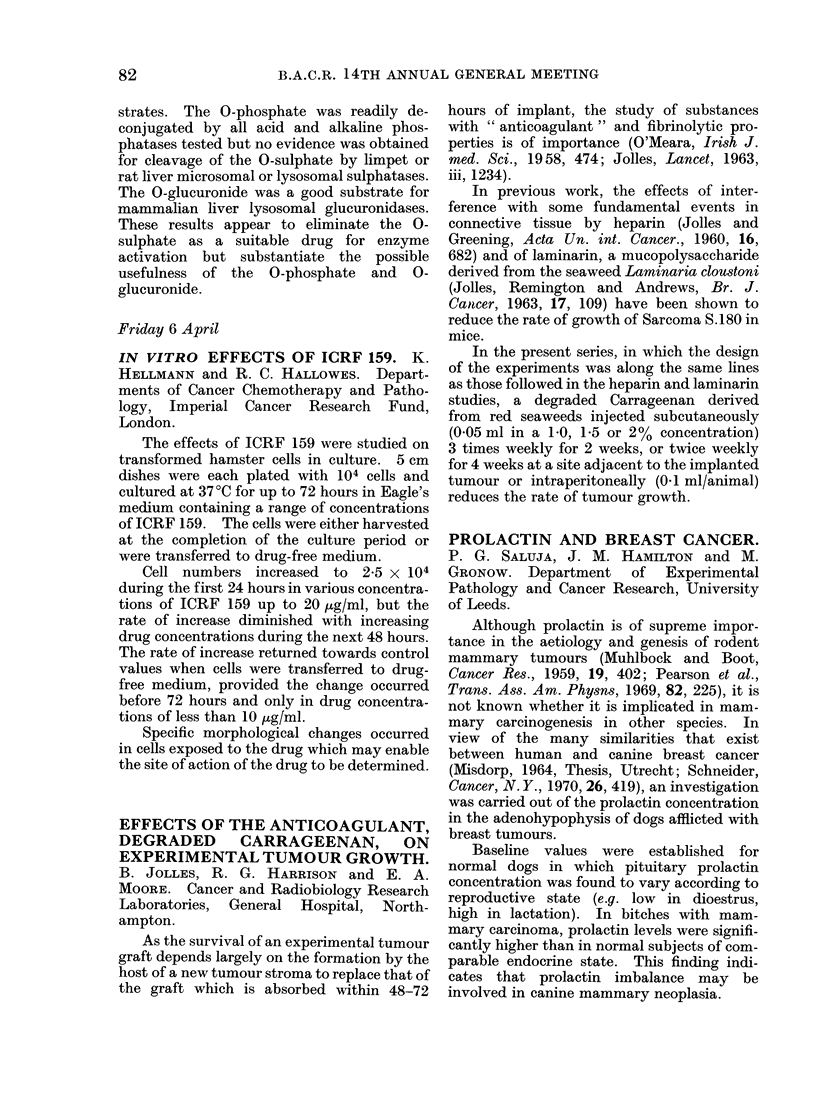# in vitro effects of ICRF 159.

**DOI:** 10.1038/bjc.1973.93

**Published:** 1973-07

**Authors:** K. Hellmann, R. C. Hallowes


*Friday* 6 *April*

**_IN VITRO_ EFFECTS OF ICRF 159.** K. HELLMANN and R. C. HALLOWES. Departments of Cancer Chemotherapy and Pathology, Imperial Cancer Research Fund, London.

The effects of ICRF 159 were studied on transformed hamster cells in culture. 5 cm dishes were each plated with $10^4$ cells and cultured at 37 °C for up to 72 hours in Eagle's medium containing a range of concentrations of ICRF 159. The cells were either harvested at the completion of the culture period or were transferred to drug-free medium.

Cell numbers increased to $2{\cdot}5 \times 10^4$ during the first 24 hours in various concentrations of ICRF 159 up to 20 $\mu$g/ml, but the rate of increase diminished with increasing drug concentrations during the next 48 hours. The rate of increase returned towards control values when cells were transferred to drug-free medium, provided the change occurred before 72 hours and only in drug concentrations of less than 10 $\mu$g/ml.

Specific morphological changes occurred in cells exposed to the drug which may enable the site of action of the drug to be determined.